# Beyond Hypothyroid Myopathy: Signal Recognition Particle (SRP)-Negative Necrotizing Dermatomyositis Unmasked by Anti-nuclear Matrix Protein 2 (Anti-NXP2) Positivity in a 60-Year-Old Woman With Hashimoto’s Thyroiditis

**DOI:** 10.7759/cureus.105765

**Published:** 2026-03-24

**Authors:** Jyothsna Goranti, Mirella Marrufo Huanca, Jose Luis Diaz Macip, Gonzalo A Banchon Macias, Zeeshan Afzal

**Affiliations:** 1 Internal Medicine, The Hospitals of Providence - Transmountain/Texas Tech University Health Sciences Center El Paso, El Paso, USA; 2 Internal Medicine/Rheumatology, Texas Tech University Health Sciences Center El Paso Paul L. Foster School of Medicine, El Paso, USA

**Keywords:** anti-nxp2, dermatomyositis, hashimoto’s thyroiditis, hypothyroid myopathy, immune-mediated necrotizing myopathy, inflammatory myopathy, malignancy screening, srp-negative myopathy

## Abstract

Necrotizing dermatomyositis (NDM) represents a rare and severe inflammatory myopathy characterized by clinical features of dermatomyositis (DM) with histopathologic evidence of myofiber necrosis and minimal inflammatory infiltrates. Immune-mediated necrotizing myopathy is classically associated with anti-signal recognition particle (SRP) and anti-HMG-CoA reductase (HMGCR) antibodies; however, seronegative cases occur and may overlap with DM-specific autoantibodies such as anti-nuclear matrix protein 2 (anti-NXP2). Coexisting metabolic conditions, including hypothyroidism, may obscure the diagnosis.

We report a 60-year-old woman with Hashimoto’s thyroiditis who developed progressive proximal weakness and markedly elevated creatine kinase (CK) levels initially attributed to uncontrolled hypothyroidism. Despite thyroid hormone optimization, weakness persisted, and a progressive erythematous rash developed. Further evaluation revealed antinuclear antibody (ANA) positivity at 1:320, positive anti-Mi-2α/β, and markedly elevated anti-NXP2 antibodies, with negative anti-SRP and anti-HMGCR antibodies. Muscle biopsy demonstrated prominent myofiber necrosis with sparse inflammatory infiltrates, confirming NDM. High-dose corticosteroids and methotrexate resulted in gradual clinical improvement. Given the anti-NXP2 positivity, comprehensive malignancy screening was initiated.

This case highlights the diagnostic challenge of distinguishing hypothyroid myopathy from inflammatory myopathy and expands the spectrum of anti-NXP2-associated disease to include SRP-negative necrotizing phenotypes. Persistent weakness or elevated CK levels despite thyroid correction should prompt evaluation for inflammatory myopathy, including myositis-specific antibodies and muscle biopsy.

## Introduction

Idiopathic inflammatory myopathies (IIMs) are a heterogeneous group of immune-mediated muscle disorders characterized by proximal muscle weakness, elevated muscle enzymes, and variable extramuscular involvement. Among these, dermatomyositis (DM) is defined by characteristic cutaneous manifestations and myositis-specific autoantibodies, whereas immune-mediated necrotizing myopathy (IMNM) is distinguished histologically by prominent myofiber necrosis with minimal inflammatory infiltrates [1].

IMNM is classically associated with anti-signal recognition particle (SRP) and anti-HMG-CoA reductase (HMGCR) antibodies. Anti-SRP-positive IMNM typically presents with acute, severe proximal weakness, markedly elevated creatine kinase (CK) levels, and a more refractory disease course. However, approximately one-third of IMNM cases are seronegative, lacking both anti-SRP and anti-HMGCR antibodies [1]. These SRP-negative variants present a diagnostic challenge and may demonstrate overlapping clinical or serologic features with DM.

Anti-nuclear matrix protein 2 (anti-NXP2) is a myositis-specific antibody traditionally associated with DM, particularly in juvenile cases, but increasingly recognized in adults [2,3]. In adult-onset disease, anti-NXP2 positivity has been associated with severe muscle involvement, dysphagia, subcutaneous edema, and increased malignancy risk [2,3]. Although primarily linked to classic DM, emerging reports describe anti-NXP2 antibodies in necrotizing phenotypes, including paraneoplastic immune-mediated necrotizing myopathy [4-6].

The coexistence of metabolic myopathy, particularly hypothyroid myopathy, may further obscure the diagnosis. Hypothyroid myopathy frequently presents with proximal weakness and significantly elevated CK levels, mimicking inflammatory myopathies [7,8]. Distinguishing between metabolic and immune-mediated etiologies is essential, especially when weakness persists despite thyroid hormone correction. Early recognition is important, as necrotizing phenotypes often require prompt and aggressive immunosuppressive therapy to prevent irreversible muscle damage [9].

We present a case of SRP-negative necrotizing dermatomyositis (NDM) associated with anti-NXP2 positivity in a patient with Hashimoto’s thyroiditis, highlighting the expanding spectrum of necrotizing variants within dermatomyositis and underscoring the importance of comprehensive evaluation in patients with persistent muscle weakness and elevated CK levels.

## Case presentation

A 60-year-old woman with a past medical history significant for Hashimoto’s thyroiditis and hypertension presented with a two-week history of progressive muscle pain, stiffness, and weakness. Symptoms initially began in the lower extremities and gradually progressed to involve the upper extremities and paraspinal musculature. She described difficulty rising from a seated position, climbing stairs, and lifting objects overhead. She denied exertional triggers, trauma, recent infections, medication changes, or statin use. There were no associated fevers, dyspnea, chest pain, dysphagia, or dark urine.

At presentation, her medications included levothyroxine 75 µg daily and chlorthalidone. She was not taking statins or other myotoxic agents.

On physical examination, she was alert and oriented. Cranial nerves II-XII were grossly intact. Neurologic examination revealed symmetric proximal muscle weakness with Medical Research Council (MRC) grade 4/5 strength in bilateral shoulder abduction, elbow flexion and extension, hip flexion, and knee flexion and extension. Distal strength was preserved, with 5/5 strength in hand grip and ankle dorsiflexion and plantarflexion. Deep tendon reflexes (biceps, triceps, patellar, and Achilles) were 2+ and symmetric. Sensation was intact to light touch and pinprick. Pathologic reflexes, including Babinski sign, were absent bilaterally.

**Figure 1:**
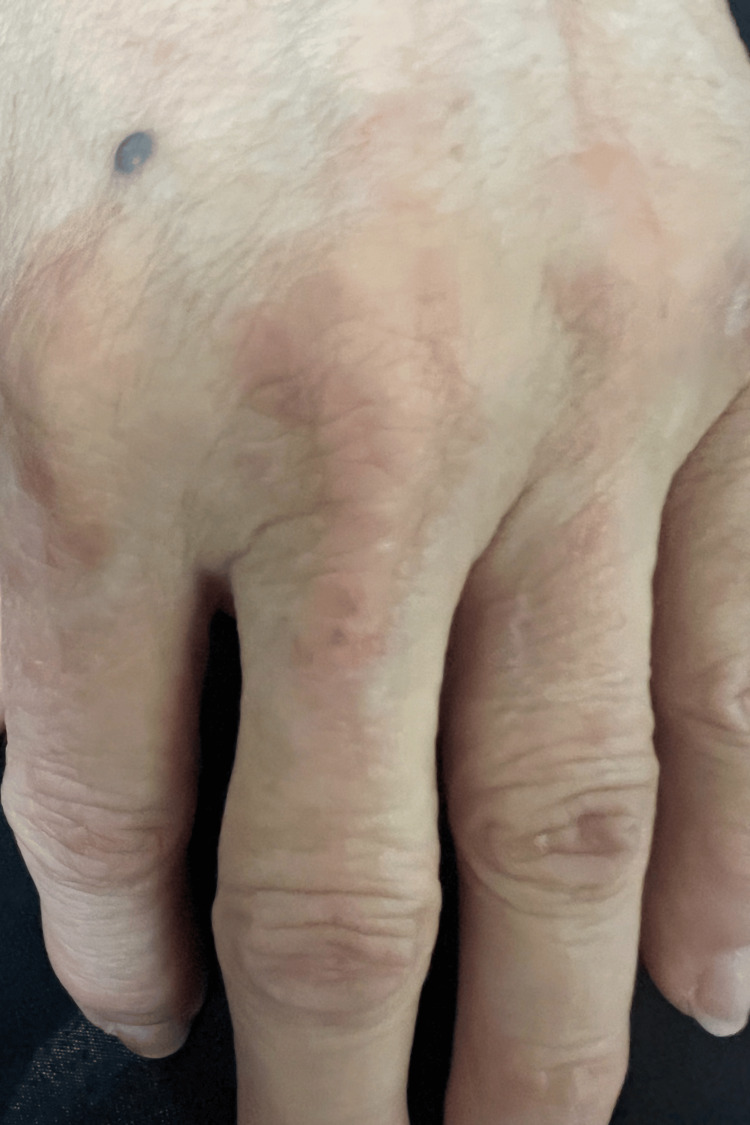
Dorsal hand demonstrating violaceous-to-erythematous macular lesions overlying the metacarpophalangeal and proximal interphalangeal joints, suggestive of Gottron-like changes associated with dermatomyositis.

**Figure 2:**
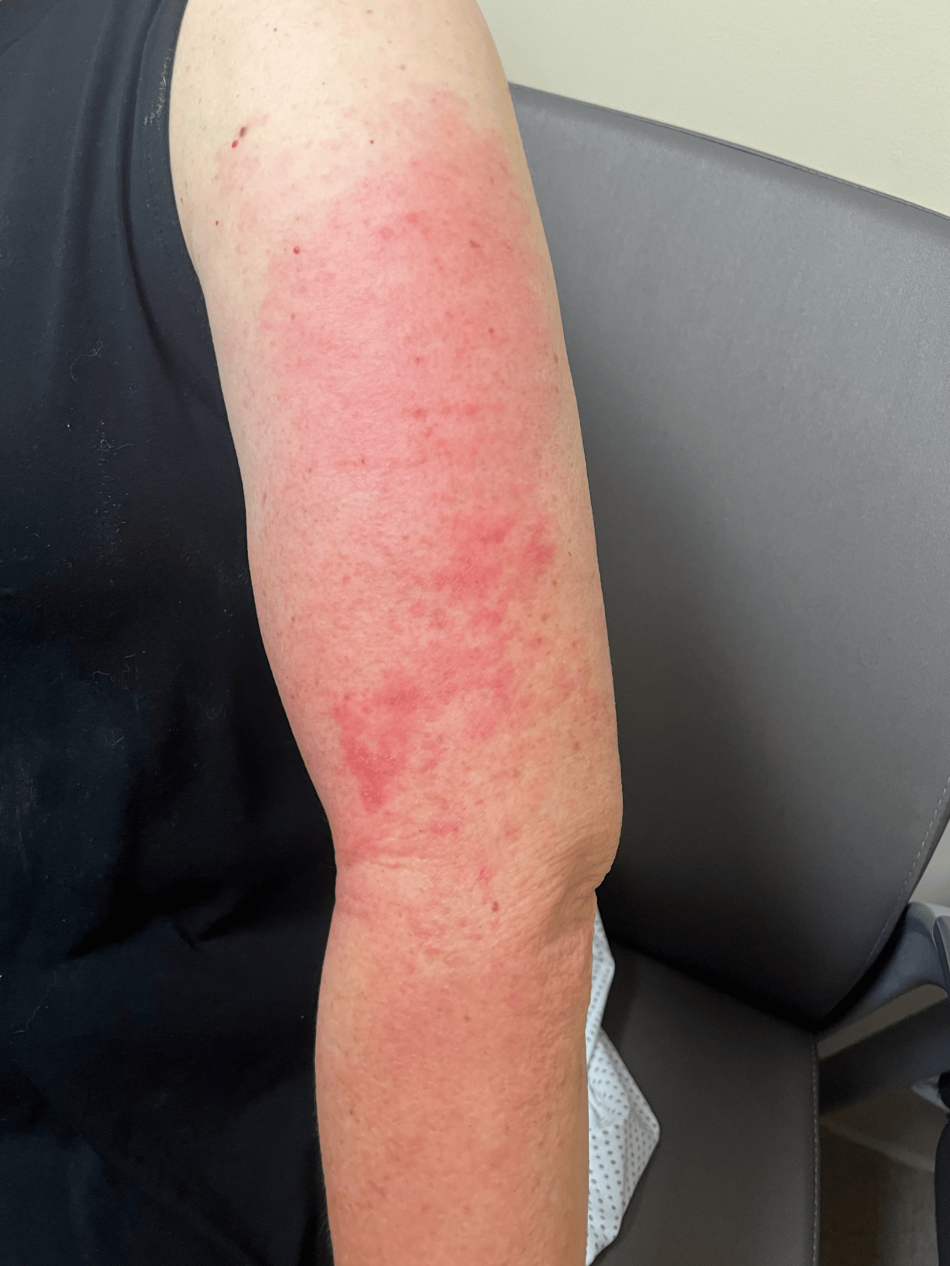
Non-blanching patchy erythematous rash involving the lateral aspect of the upper arm, consistent with cutaneous manifestations of dermatomyositis.

Musculoskeletal examination revealed diffuse upper extremity tenderness to palpation, with pain more pronounced during active than passive range of motion. Dermatologic examination demonstrated non-blanching erythematous patches over the left forehead and dorsal hands, along with patchy erythema of the upper extremities, without vesiculation, scaling, or crusting (Figures 1, 2).

Initial laboratory evaluation is summarized in Table 1. The patient demonstrated markedly elevated muscle enzymes in the setting of elevated thyroid-stimulating hormone, preserved renal function, and minimal inflammatory marker elevation.

**Table 1:** Initial laboratory evaluation at presentation.

Laboratory Test	Result	Reference Range	Interpretation
Creatine kinase (CK)	5,498 U/L	30–200 U/L	Markedly elevated
Aldolase	30.2 U/L	1.0–7.5 U/L	Elevated
Thyroid-stimulating hormone (TSH)	12 µIU/mL	0.4–4.5 µIU/mL	Elevated
Free T4	0.9 ng/dL	0.8–1.8 ng/dL	Low-normal
Creatinine	0.6 mg/dL	0.6–1.3 mg/dL	Normal
Erythrocyte sedimentation rate (ESR)	11 mm/hr	0–20 mm/hr	Normal
C-reactive protein (CRP)	0.9 mg/dL	<1.0 mg/dL	Normal
Urinalysis	Negative for myoglobin	—	No myoglobinuria
Complete blood count (CBC)	Unremarkable	—	Normal
Comprehensive metabolic panel (CMP)	Unremarkable	—	Normal

Given the markedly elevated CK in the setting of elevated TSH and preserved renal function, the initial impression was rhabdomyolysis secondary to hypothyroid myopathy.

She was admitted for intravenous hydration, pain control with acetaminophen and ketorolac, and serial monitoring of CK and electrolytes. An autoimmune workup was initiated, including ANA, anti-Jo-1, anti-Mi-2, anti-Ro, anti-La, ESR, CRP, and aldolase. Levothyroxine was increased from 75 µg to 100 µg daily. She remained hemodynamically stable and was discharged with instructions for hydration and outpatient follow-up. At discharge, proximal muscle strength remained 4/5 in the upper and lower extremities, with mild functional improvement. The working diagnosis at discharge was hypothyroid myopathy.

Two weeks later, she returned with persistent proximal muscle weakness and pain, now predominantly affecting the upper extremities and neck. She also reported progression of the erythematous, pruritic rash over the left forehead and forearm, noting that the rash had preceded the onset of muscle symptoms. She denied Raynaud’s phenomenon, photosensitivity, oral ulcers, or inflammatory joint symptoms.

Repeat laboratory evaluation and extended myositis-specific antibody testing are summarized in Table 2.

**Table 2:** Follow-up laboratory and autoimmune evaluation.

Category	Laboratory Test	Result	Interpretation
Dermatomyositis-related antibodies	Antinuclear antibody (ANA)	Positive, 1:320 (nuclear speckled pattern)	Positive
	Anti-nuclear matrix protein 2 (anti-NXP2) antibody	139 U	Markedly elevated
	Anti-Mi-2 alpha antibody	28 U	Positive
	Anti-Mi-2 beta antibody	139 U	Positive
	Anti-melanoma differentiation-associated gene 5 (anti-MDA5) antibody	Negative	Not detected
	Anti-transcription intermediary factor 1-gamma (anti-TIF1-γ) antibody	Negative	Not detected
Other autoimmune markers	Centromere protein B (CENP-B) antibody	54 U	Positive
	Anti-double-stranded DNA (anti-dsDNA) antibody	Negative	Not detected
	Anti-smooth muscle antibody	Negative	Not detected
	Anti-topoisomerase I (anti–Scl-70) antibody	Negative	Not detected
	Anti-Sjogren’s syndrome A (anti-SSA/Ro) antibody	Negative	Not detected
	Anti-Sjogren’s syndrome B (anti-SSB/La) antibody	Negative	Not detected
	Anti-cyclic citrullinated peptide (anti-CCP) antibody	Negative	Not detected
	Rheumatoid factor	Negative	Not detected
	Anti-histidyl-transfer RNA synthetase (anti–Jo-1) antibody	<0.2	Negative
Muscle enzymes and thyroid function	Creatine kinase	974 U/L (reference range: 30–200 U/L)	Elevated
	Aldolase	17.3 U/L (reference range: 1.0–7.5 U/L)	Elevated
	Thyroid-stimulating hormone (TSH)	3.19 µIU/mL (reference range: 0.4–4.5 µIU/mL)	Normalized
Infectious screening	Hepatitis C antibody	Reactive	Prior exposure suspected
	Hepatitis C virus RNA	Negative	No active infection

Despite improvement in thyroid function, CK remained elevated and proximal weakness persisted, raising concern for inflammatory myopathy. Computed tomography (CT) imaging of the chest, abdomen, and pelvis revealed no acute findings.

A left deltoid muscle biopsy was performed. Histopathologic examination demonstrated moderate fiber size variability with scattered degenerating and necrotic fibers, along with myophagocytosis. Internalized nuclei were mildly increased. Inflammatory infiltrates were present around blood vessels and focally surrounding individual myofibers. There was no evidence of perifascicular atrophy, group atrophy, vasculitis, or fibrinoid necrosis, and endomysial connective tissue was not increased.

Histochemical staining demonstrated preserved myofibrillar architecture on trichrome staining. NADH staining demonstrated a normal intermyofibrillar network. ATPase stains showed preserved fiber type distribution with a checkerboard pattern, and esterase staining confirmed the absence of angulated fibers.

Overall, the findings were consistent with necrotizing inflammatory myopathy. In the context of negative anti-SRP and anti-HMGCR antibodies and positive anti-NXP2 antibodies, these findings supported a diagnosis of SRP-negative NDM.

She was initiated on prednisone 60 mg daily. Methotrexate 15 mg weekly with folic acid 1 mg daily was added as a steroid-sparing agent and later titrated to 20 mg weekly. Prednisone was gradually tapered to 40 mg and then 30 mg daily. Levothyroxine dosage was adjusted back to 75 µg daily following normalization of thyroid function.

Given the presence of anti-NXP2 antibodies and their known association with malignancy risk, comprehensive malignancy screening was undertaken. Colonoscopy and screening mammography were normal. Gynecologic evaluation was recommended, and CT imaging showed no evidence of occult malignancy.

She was referred for physical therapy twice weekly for strength rehabilitation. At the most recent follow-up, she demonstrated gradual improvement in proximal muscle strength and mobility, along with stabilization of CK and aldolase levels.

## Discussion

This case underscores the evolving understanding of necrotizing variants within the spectrum of DM and IMNM. IMNM is increasingly recognized as a distinct subgroup of IIMs, characterized by prominent myofiber necrosis with minimal inflammatory infiltrates and typically associated with anti-SRP or anti-HMGCR antibodies [1,10]. However, up to one-third of patients with necrotizing inflammatory myopathy remain seronegative for these classic antibodies [1,11]. The SRP-negative variant poses diagnostic complexity, particularly when overlapping DM-specific antibodies are present.

Anti-SRP-positive IMNM is classically associated with rapidly progressive symmetric proximal weakness, markedly elevated CK levels (often >5,000 U/L), treatment resistance, and limited extramuscular manifestations [10]. In contrast, SRP-negative IMNM demonstrates greater phenotypic heterogeneity and may exhibit cutaneous findings, overlap autoantibodies, and variable therapeutic response [1,11]. This blurs the traditional distinction between IMNM and DM, supporting the concept that these entities exist along a clinicopathologic spectrum rather than as rigidly separate categories.

In our patient, muscle biopsy demonstrated myofiber necrosis, degenerating fibers, and myophagocytosis with relatively sparse inflammatory infiltrates, hallmarks of necrotizing myopathy [1,10]. However, anti-SRP and anti-HMGCR antibodies were negative, and anti-NXP2 antibodies were strongly positive. The presence of DM-associated cutaneous manifestations further supports classification as an SRP-negative NDM phenotype rather than classic IMNM.

Anti-NXP2 and phenotypic implications

Anti-NXP2 antibodies define a clinically distinct subset of DM. In adults, anti-NXP2 positivity has been associated with severe muscle involvement, dysphagia, subcutaneous edema, and increased malignancy risk [2,5]. Large cohort studies demonstrate a higher incidence of cancer in anti-NXP2-positive adults compared with antibody-negative DM patients [2,5]. The most commonly reported associated malignancies include gastrointestinal cancers (particularly colorectal), genitourinary cancers, and lung cancers [12,13]. Retrospective analyses further suggest that anti-NXP2 positivity correlates with extensive muscle necrosis on biopsy and more aggressive myopathic features [3,4].

Notably, anti-NXP2 antibodies have also been reported in patients with necrotizing myopathy patterns, including paraneoplastic presentations [4,6]. This observation reinforces the concept that myositis-specific antibodies may define immunologic phenotypes that transcend strict histopathologic boundaries [1,12]. Therefore, the combination of necrotizing biopsy findings and anti-NXP2 positivity in this case likely represents an overlap phenotype within the DM-IMNM spectrum.

Malignancy risk

Malignancy risk remains a critical consideration in DM, particularly in patients with myositis-specific antibodies such as anti-NXP2 or anti-TIF1-γ [2,5,13]. Population-based studies confirm an increased standardized incidence ratio for malignancy in adult DM, especially within the first three to five years following diagnosis [13]. Given this established association, comprehensive age-appropriate cancer screening and ongoing surveillance are recommended [2,5,13]. Our patient appropriately underwent malignancy evaluation following identification of anti-NXP2 positivity.

Diagnostic complexity: hypothyroid myopathy mimic

The presence of uncontrolled hypothyroidism significantly complicated the initial diagnostic evaluation. Hypothyroid myopathy can present with proximal weakness and markedly elevated CK levels, occasionally exceeding 5,000 U/L, closely mimicking inflammatory myopathy [7,8]. However, hypothyroid myopathy typically improves with restoration of euthyroid status [7]. In this case, persistence of weakness and progression of rash despite normalization of thyroid function served as critical diagnostic clues favoring inflammatory myopathy.

Recent genetic and epidemiologic studies also suggest a bidirectional association between thyroid dysfunction and inflammatory myopathies, further complicating clinical interpretation [8]. Thus, the coexistence of thyroid disease does not exclude inflammatory myopathy and may delay diagnosis if relied upon prematurely.

Therapeutic implications

Necrotizing phenotypes frequently require early aggressive immunosuppressive therapy to prevent irreversible muscle damage [9,14]. Current management strategies recommend high-dose corticosteroids combined with a steroid-sparing immunosuppressive agent such as methotrexate or azathioprine [9,14]. Intravenous immunoglobulin has demonstrated efficacy in refractory or severe cases and may be considered early in aggressive necrotizing presentations [9].

Anti-SRP-positive disease has been associated with greater treatment resistance and need for combination or biologic therapy [10]. In contrast, SRP-negative variants may demonstrate more favorable responses to conventional regimens, though data remain limited [1,11]. Our patient exhibited gradual clinical and biochemical improvement with corticosteroids and methotrexate, supporting the responsiveness of this SRP-negative NDM phenotype.

Clinical significance

Recognition of SRP-negative NDM is clinically important for several reasons. Absence of anti-SRP and anti-HMGCR antibodies does not exclude necrotizing inflammatory myopathy, and comprehensive myositis-specific antibody panels are essential for accurate classification [1,11]. Anti-NXP2 positivity warrants structured and ongoing malignancy surveillance given the association with increased cancer risk [2,5,13]. Necrotizing variants frequently require early combination immunosuppressive therapy to prevent irreversible muscle damage [9,14]. Furthermore, overlapping serologic and histopathologic features support a spectrum-based model of inflammatory myopathies rather than rigid categorical distinctions [1,12].

Overall, this case illustrates that SRP-negative NDM may present with overlapping immunologic and morphologic characteristics. Accurate classification requires integration of clinical presentation, serologic profile, and muscle biopsy findings to guide timely therapy and malignancy surveillance.

## Conclusions

SRP-negative NDM is an uncommon and diagnostically challenging entity within the inflammatory myopathy spectrum. The absence of anti-SRP antibodies does not exclude necrotizing inflammatory myopathy, particularly when DM-specific antibodies such as anti-NXP2 are present. Anti-NXP2 positivity is associated with an increased risk of malignancy, often in the context of a paraneoplastic phenomenon rather than a direct causal risk factor for a specific cancer. This association warrants comprehensive evaluation and ongoing surveillance.

Persistent proximal weakness and elevated CK levels despite correction of hypothyroidism should prompt investigation for inflammatory myopathy, including extended antibody testing and muscle biopsy. Early recognition of SRP-negative necrotizing variants is essential to initiate appropriate immunosuppressive therapy and improve clinical outcomes.

## References

[1] Kamperman RG, van der Kooi AJ, de Visser M, Aronica E, Raaphorst J (2022). Pathophysiological mechanisms and treatment of dermatomyositis and immune mediated necrotizing myopathies: a focused review. Int J Mol Sci.

[2] Rogers A, Chung L, Li S, Casciola-Rosen L, Fiorentino DF (2017). Cutaneous and systemic findings associated with nuclear matrix protein 2 antibodies in adult dermatomyositis patients. Arthritis Care Res (Hoboken).

[3] Xie X, Dai X, Liu H, Xing Y (2024). A retrospective study for clinical characteristics of 293 patients with dermatomyositis. Medicine (Baltimore).

[4] Ichimura Y, Konishi R, Shobo M (2022). Anti-nuclear matrix protein 2 antibody-positive inflammatory myopathies represent extensive myositis without dermatomyositis-specific rash. Rheumatology (Oxford).

[5] Albayda J, Pinal-Fernandez I, Huang W (2017). Antinuclear matrix protein 2 autoantibodies and edema, muscle disease, and malignancy risk in dermatomyositis patients. Arthritis Care Res (Hoboken).

[6] Su L, Yang Y, Jia Y, Liu X, Zhang W, Yuan Y, Li Z (2018). Anti-NXP2-antibody-positive immune-mediated necrotizing myopathy associated with acute myeloid leukemia: a case report. Medicine (Baltimore).

[7] Fariduddin MM, Haq N, Bansal N (2025). Hypothyroid myopathy. StatPearls [Internet].

[8] Li Q, Yang S, Ma Y, Huang H, Zhi L, Wang S, Lu L (2024). Hypothyroidism and dermato/polymyositis: a two-sample Mendelian randomization study. Front Endocrinol (Lausanne).

[9] Suh J, Amato AA (2024). Management of immune-mediated necrotizing myopathy. Muscle Nerve.

[10] Allenbach Y, Mammen AL, Benveniste O, Stenzel W (2018). 224th ENMC International Workshop: clinico-sero-pathological classification of immune-mediated necrotizing myopathies Zandvoort, The Netherlands, 14-16 October 2016. Neuromuscul Disord.

[11] Pinal-Fernandez I, Parks C, Werner JL (2017). Longitudinal course of disease in a large cohort of myositis patients with autoantibodies recognizing the signal recognition particle. Arthritis Care Res (Hoboken).

[12] Betteridge Z, McHugh N (2016). Myositis-specific autoantibodies: an important tool to support diagnosis of myositis. J Intern Med.

[13] Hill CL, Zhang Y, Sigurgeirsson B (2001). Frequency of specific cancer types in dermatomyositis and polymyositis: a population-based study. Lancet.

[14] Dalakas MC (2015). Inflammatory muscle diseases. N Engl J Med.

